# A persistent epidemic of porcine epidemic diarrhoea virus infection by serological survey of commercial pig farms in northern Vietnam

**DOI:** 10.1186/s12917-021-02941-7

**Published:** 2021-07-05

**Authors:** Ohnmar Myint, Nguyen Thi Hoa, Naoyuki Fuke, Apisit Pornthummawat, Nguyen Thi Lan, Takuya Hirai, Ayako Yoshida, Ryoji Yamaguchi

**Affiliations:** 1 Department of Veterinary Pathology, Faculty of Agriculture, University of Miyazaki, 1-1 Gakuenkibanadai-Nishi, 889-2192 Miyazaki, Japan; 2 Vietnam National University of Agriculture, Gia Lam, Hanoi, Vietnam; 3 Department of Veterinary Parasitic Diseases, Faculty of Agriculture, University of Miyazaki, Miyazaki, Japan; 4 Center for Animal Disease Control, University of Miyazaki, Miyazaki, Japan

**Keywords:** Porcine epidemic diarrhoea virus, PED, Serological survey, Pig farm, Northern Vietnam

## Abstract

**Background:**

Porcine epidemic diarrhoea (PED) is a highly contagious infectious disease with negative economic impacts on the swine industry. PED outbreaks were reported from 2009 to 2015, but sporadic infection has been observed until now in Vietnam. However, the seroprevalence of PEDV infection has not yet been reported for commercial pig farms in Vietnam. The aim of this study was to assess the seroprevalence of PEDV infection in Vietnamese pig farms to reveal the endemic status of PEDV in northern Vietnam.

**Results:**

A serological survey of PEDV infection was carried out using indirect ELISA in commercial pig farms in Hai Duong, Hung Yen and Thai Binh provinces in northern Vietnam in 2019. Twenty sera were randomly collected from each of 10 commercial pig farms, from each province; none of the farms had vaccinated for PEDV. Serological evidence of natural PEDV infection, expressed as a high antibody titre, was observed in the pig farms in all 3 provinces. The OD values were significantly higher (p < 0.001) for pig sera from Thai Binh than from Hai Duong and Hung Yen. No significant differences (p > 0.05) were detected for seropositivity to PEDV based on locality, age, pig breed and farm size.

**Conclusions:**

This study indicates serological evidence of natural PEDV infection with high antibody titre in commercial pig farms. PEDV infection was widespread among the pig population in these 3 provinces and that good management and strict biosecurity are needed at these pig farms.

## Background

Porcine epidemic diarrhoea (PED) is a highly contagious infectious disease characterised by watery diarrhoea and vomiting that leads to dehydration and high mortality, especially in suckling piglets. Consequently, PED outbreaks cause substantial economic losses to the swine industry [1, 2]. PED is caused by PED virus (PEDV), which belongs to order *Nidovirales*, family *Coronaviridae*, genus *Alphacoronavirus* [3, 4].

During the 1970 and 1980s, PEDV infection was widespread in Europe [5]. In Asia, PED was found in Japan, China, South Korea and Thailand [6–9]. Since 2013, infection with a highly virulent PEDV strain has occurred in the United States and then spread to Canada, Mexico, Japan, South Korea, Thailand, Taiwan and the Philippines [10–16]. PEDV infection has now become endemic and has a strong negative economic impact on the swine industry worldwide.

In Vietnam, a PED outbreak in piglets was first reported in 2009 [17] and a new variant PEDV infection occurred in 2013 [18]. Other researchers have described that heterogeneous PEDV strains, including a US-like strain, were found in northern Vietnam from 2012 to 2015 [19]. Sporadic PEDV infection is still currently present; however, the prevalence of PEDV infection in commercial pig farms in Vietnam remains unknown. We established the enzyme linked immunosorbent assay (ELISA) for PEDV specific antibody detection in previous study [20]. The aim of the present study was to determine the seroprevalence of PEDV infection in pig farms to reveal the endemic status of PEDV in northern Vietnam by applying the developed ELISA protocol under field conditions. The findings of this study will be helpful in the prevention and control of PED in Vietnam.

## Results

### Clinical survey for PEDV infection in northern Vietnam

Only two of the 30 pig farms had active ongoing diarrhoea during sera collection; one farm was in Hung Yen and the other was in Thai Binh. None of the other 28 pig farms showed diarrhoea symptoms during sera collection.

### The proportion of PEDV seropositivity based on locality

Seropositivity to PEDV infection was detected in 9 of 10 (90 %) commercial pig farms in Hai Duong, in 10 of 10 (100 %) farms in Hung Yen and in 10 of 10 (100 %) farms in Thai Binh. The overall PEDV seropositivity for the 3 provinces was 96.7 %. The proportion of PEDV seropositive farms based on locality did not differ significantly (P > 0.05) between the three provinces (Table 1).

**Table 1:** Proportion of PEDV seropositive farms and pigs based on locality

	No. of seropositive farms or pigs/No. examined farms or pigs		
Location	Hai Duong	Hung Yen	Thai Binh
Farms	9/10	10/10	10/10
No. of pigs	172/200	178/200	183/200

 A total of 200 pig sera were collected from each province. The percentage of PEDV seropositive pigs was 86 % (172/200 pigs) in Hai Duong, 89 % (178/200 pigs) in Hung Yen and 91.5 % (183/200 pigs) in Thai Binh. These proportions of PEDV seropositive pigs based on locality did not differ significantly (*P* > 0.05) between the 3 provinces (Table 1).

**Fig. 1:**
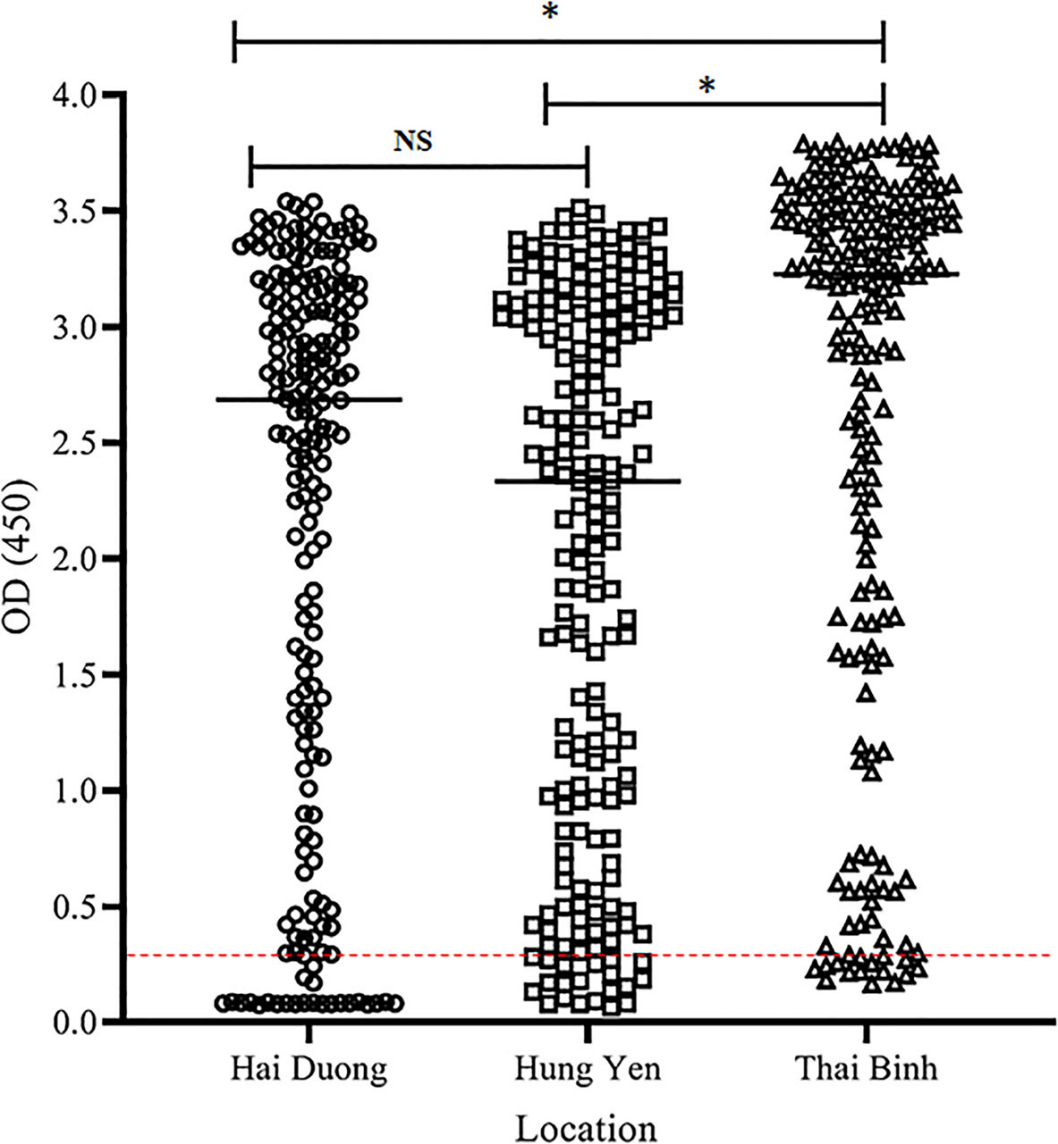
Reactivity of pig sera from three provinces to PED whole viral antigen in indirect ELISA. The asterisk indicates the significant difference based on one-way ANOVA test. (*, *P* < 0.001; NS, not significant). The dotted line indicates the cut-off value (0.320) of the ELISA

The serum OD values were significantly higher for samples from Thai Binh than from Hai Duong and Hung Yen (P < 0.001). However, the serum OD values were not significantly different between Hai Duong and Hung Yen (P > 0.05). The OD values of all swine sera from the 3 provinces are shown in Fig. 1.

### The analysis for number of detected pigs based on OD value

The serum OD values were classified into five groups: less than 0.320 (seronegative), 0.320–1.0, 1.1–2.0, 2.1–3.0 and over 3.1. The number of pigs was significantly larger in the group with OD value over 3.1 than in the groups with OD value less than 0.320, 0.320–1.0 and 1.1–2.0 (*P* < 0.05). However, no significant differences (*P* > 0.05) were noted in the number of pigs in the group with OD values over 3.1 and the OD values 2.1–3.0 group. The differences between the number of pigs in the groups with OD values of less than 0.320, 0.320–1.0, 1.1–2.0 and 2.1–3.0 were not significantly different (*P* > 0.05). The analysis of the numbers of seropositive pigs and OD values is shown in Table 2.

**Table 2:** Analysis between the numbers of examined pigs based on OD value

Location	OD values				
	< 0.320	0.320–1.0	1.1–2.0	2.1–3.0	≥ 3.1
Hai Duong	28	19	24	64	65
Hung Yen	22	37	28	60	53
Thai Binh	17	18	21	29	115
Total	67^a^	74^a^	73^a^	153^ab^	233^b^

^a,b,^ The data within the same row with the different superscripts are significantly different at *P*<0.05.

### The PEDV seropositivity based on age groups

**Fig. 2:**
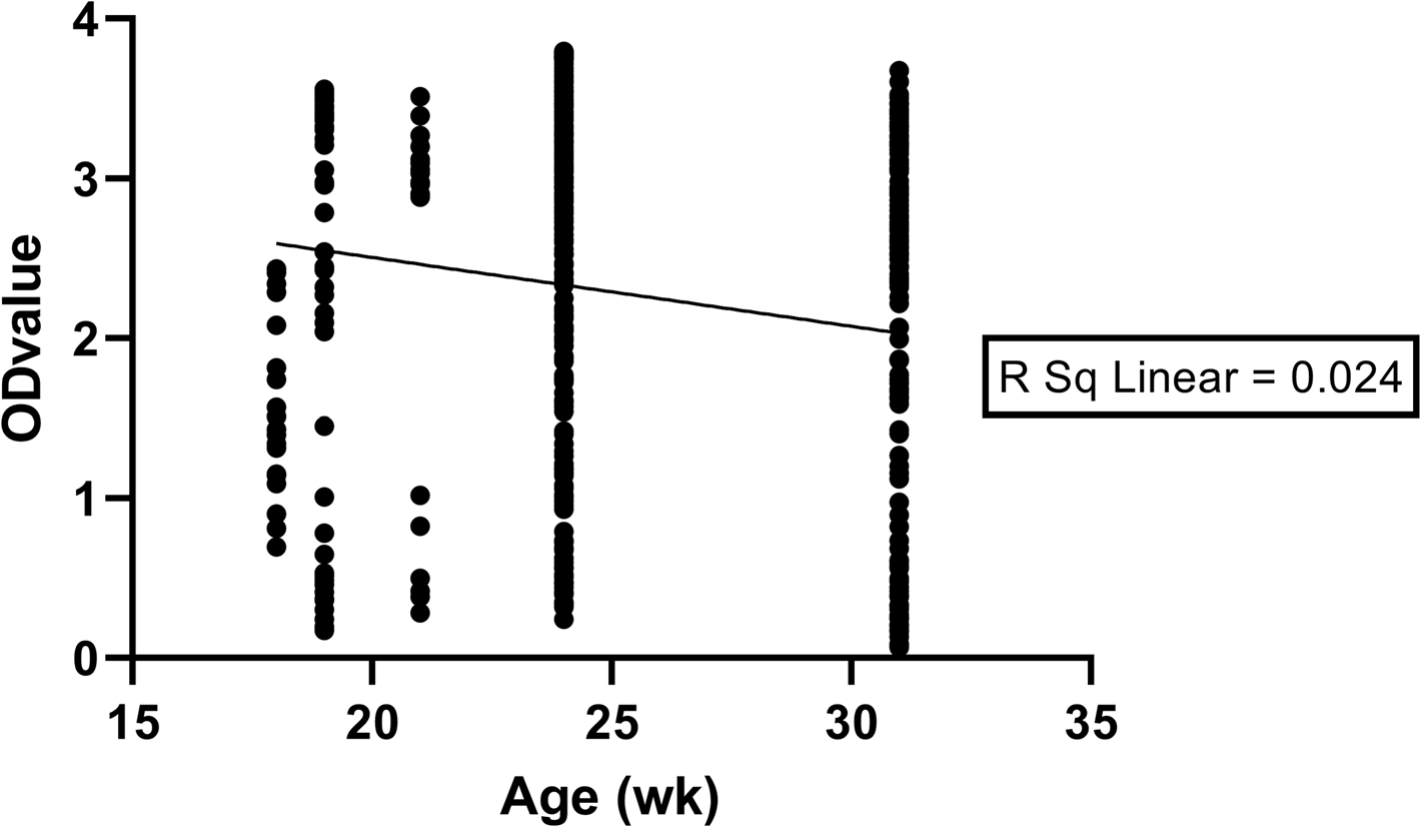
Correlation between pig age and OD value

The collected pig sera were divided into 3 age groups: 18–23 weeks, 24–30 weeks and over 31 weeks of age. In all provinces, the percentage of PEDV seropositive farms were 100 %, 100% and 90.9 % for the 18–23, 24–30 and over 31 weeks age groups, respectively. No significant differences (*P* > 0.05) were found in the proportion of PEDV seropositive farms in terms of the 3 age groups. A very weak negative, but statistically significant, correlation was detected between the pig age and the serum OD values (*r* = − 0.155, *P* < 0.0001). The corelation between the pig age and their serum OD values are shown in Fig. 2.

### The PEDV seropositivity based on the pig breeds

The percentage of PEDV seropositive farms in the 3 provinces was 100% and 92.9 % for the LYD and LY pig breeds, respectively, and was not significantly different (*P* > 0.05).

### The PEDV seropositivity based on farm sizes

 The percentage of PEDV seropositive farms in the 3 provinces was 100 %, 100 %, and 93.3 % when farms were classified according to farm size with pig numbers of less than 1000, 1001–1300 and over 1301 pigs per farm, respectively. No significant differences (*P* > 0.05) were detected in the proportion of PEDV seropositive farms according to farm size. The analysis of PEDV seropositive farms based on age, breed and farm size is presented in Table 3.

**Table 3:** Proportion of PEDV seropositive farms based on age, breed groups and farm size

	Criteria	No. of PEDV seropositive farms/No. of examined farms
Age	18–23 weeks	5/5
	24–30 weeks	14/14
	Over 31 weeks	10/11
Breed	LYD	16/16
	LY	13/14
Farm size (no. of pigs)	Less than 1000	6/6
	1001–1300	9/9
	Over 1301	14/15

## Discussion

The indirect ELISA for detection of PEDV-specific antibody developed in a previous study has high sensitivity and specificity for disease monitoring and control. The ELISA test can apply for detection on specific antibody IgG of all PEDV strains and the sensitivity and specificity were 92.6 and 90.1 %, respectively [20]. The ELISA tests are cost effective, simple, rapid, and accurate and can evaluate many serum samples at the same time. This study was conducted to evaluate the endemic status of PED in northern Vietnam by applying the developed ELISA protocol under field conditions.

The serological survey of PEDV infection was carried out in commercial pig farms that had not vaccinated for PEDV in the 3 provinces of northern Vietnam to establish monitoring and disease control management strategies. Serological evidence of PEDV infection was widespread in the commercial pig farms of these 3 provinces, indicating that the PEDV seropositivity in the pig farms was due to natural infection.

No significant differences were found for the percentage of PEDV seropositive pig farms in Hai Duong, Hung Yen and Thai Binh provinces because almost every pig farm showed PEDV seropositivity. The numbers of PEDV seropositive pigs from the 3 provinces were also not significantly different between the provinces. These data again indicated that PEDV infection was naturally widespread in the pig populations in these 3 provinces. In other report, 30.89 % of pig farms were found as PED positive in northern Vietnam which was detected by LAMP method and these farms at northern Vietnam may be the source of transmission in countrywide [21].

A very high PEDV-specific ELISA signal was observed in many PEDV seropositive pigs (Fig. 1). The number of PEDV seropositive pigs that showed OD values above 3.1 was higher than the number of PEDV seropositive pigs that had OD values less than 2 (Table 2). The OD values were also significantly higher for pig sera from Thai Binh than from the other 2 provinces. These higher OD values indicate that PEDV infection has likely been occurring continuously in these pig farms. The pigs with 8 to 12-week-old did not show any clinical signs of PED, even though they showed a high antibody titre following experimental PEDV infection [22]. PEDV infection can also cause subclinical infections with no clinical signs in finishing pigs, and these asymptomatic pigs could therefore represent an important risk factor for spreading the disease [23]. It was reported that 70 % of PED positive farms were subclinically infected in Northern Vietnam and this may be the risk of cross-contamination in pigs in Vietnam [21]. In the present study, symptoms such as diarrhoea were not observed at most of the pig farms. Consequently, asymptomatic pigs might be contributing to the high endemic prevalence of PEDV in Vietnam.

Pigs at 18 weeks to over 31 weeks of age were analysed by correlation analysis with their serum OD values. The very weak negative correlation observed between age and OD values suggests that the younger pigs had a higher serum OD values. This means that PED infection would occur recently before serum collection and may also be persistent infection in these farms. The antibody titre may be lower in older pigs after peak level of antibody had already been achieved [22].

PEDV infects pigs at all ages and causes poor performance in the feed conversion ratio and average daily weight gain of growing pigs [2, 24]. The risk factors for PED outbreaks in Vietnam were as poor biosecurity regarding fomites and, vehicle, animal, and human traffic on the farms [17]. The observed PEDV seropositivity based on age, pig breed and farm size did not differ significantly among the pig farms from the 3 provinces. These data suggest that PEDV can infect LYD and LY crossbreed pigs of all ages and that PEDV infection was observed on small as well as large farms. Many infections are probably be due to poor management and improper biosecurity on the farms.

## Conclusions

The findings of this study provide serological evidence of natural PEDV infection with high antibody titre in 3 provinces in northern Vietnam. Subclinical infection should be considered as a risk for the broad and permanent circulation of PEDV in this region. Further studies should be carried out to elucidate the extent of the subclinical infection and epidemiological surveillance should be initiated at pig farms to implement proper PED control management.

## Methods

### Study area and serological detection for PEDV infection

This serological survey was carried out at commercial pig farms in Hai Duong, Hung Yen and Thai Binh provinces of northern Vietnam from September to December, 2019. Detection of PEDV-specific antibody was conducted at Vietnam National University of Agriculture, Hanoi, Vietnam. These pigs were not aggressively operated for sample collection. The farm owners sometimes requested to check highly pathogenic porcine respiratory syndrome virus (HP-PRRSV), classical swine fever virus (CSFV) and porcine epidemic diarrhoea virus (PEDV) antibodies titre in pigs of their farms for health check. Although serological tests for PRRS and CSF were established, serological test for PED has not established yet in the Vietnam laboratory. The permission for sample collection from farm owners has already obtained according to their requests. As we established the ELISA [20], we applied it to detect the PEDV specific antibody detection. And then, we reported the endemic status of PED at Vietnam. The ELISA plates for PED detection were prepared at the University of Miyazaki, Japan, as previously described [20], and kept at 4 °C until used.

### The collection of pig blood samples for PEDV infection survey

 Ten commercial pig farms were randomly chosen in each of the 3 provinces of northern Vietnam. The twenty pig sera were randomly collected from each farm, which had not used PED vaccines. The age of the pigs was ranged from 18 to over 31 weeks. The pig breeds were a Landrace and Yorkshire crossbreed (LY) and a Landrace, Yorkshire and Duroc crossbreed (LYD). The farm pig populations ranged from 500 to 2386 pigs. All sera were collected from pig farms that used vaccines for foot and mouth disease, porcine respiratory and reproductive syndrome, classical swine fever and *Mycoplasma hyopneumoniae* infections.

### Serum preparation

A total of 600 blood samples were aseptically collected from the pigs via the jugular vein. Sera were separated by centrifugation and stored at -20 °C until used.

### Serological method

The PEDV-specific antibody was detected by indirect ELISA, as previously described [20]. PEDV NK94P6 strain was used as coated antigen in this ELISA. This strain is gold standard strain in Japan and involved in classical clade of group 1 [20, 23]. After 20 min incubation of substrate solution, the reaction was stopped and the ELISA plates were read at 450 nm in a plate reader (Epoch ^TM^ 2, BioTek® Instruments, Inc., USA) with an optical density (OD) cut-off value of 0.320.

### Data analysis

 The data were analysed for seropositivity to PEDV based on locality, age, pig breed and farm size with the Chi-square test. The age of pigs and their OD values were analysed by correlation analysis. The number of pigs with specific optical density (OD) values and the OD values of all sera based on the 3 provinces were analysed by one-way ANOVA and post hoc test using GraphPad Prism 8 software.

## Data Availability

The data supporting the conclusions of this case report are included in this article. All data sets can be requested from correspondence with the authors.

## Abbreviations

ELISA: Enzyme-linked immunosorbent assay
LYD: Landrace, Yorkshire and Duroc crossbreed
LY: Landrace and Yorkshire crossbreed
OD: Optical density
PED: Porcine epidemic diarrhoea
